# Massive eosinophilia despite severe aplastic anemia

**DOI:** 10.1002/jha2.458

**Published:** 2022-05-04

**Authors:** Aya Takahashi, Yoshihito Iwahara, Hisanori Machida, Keishi Naruse, Eiji Takeuchi, Tsutomu Shinohara

**Affiliations:** ^1^ Division of Dermatology National Hospital Organization Kochi Hospital Kochi Japan; ^2^ Division of Internal Medicine National Hospital Organization Kochi Hospital Kochi Japan; ^3^ Division of Allergy National Hospital Organization Kochi Hospital Kochi Japan; ^4^ Division of Pathology National Hospital Organization Kochi Hospital Kochi Japan; ^5^ Department of Clinical Investigation National Hospital Organization Kochi Hospital Kochi Japan; ^6^ Department of Community Medicine for Respirology Graduate School of Biomedical Sciences Tokushima University Tokushima Japan

1

A 47‐year‐old nonsmoking man developed very severe aplastic anemia (AA) (hemoglobin 56 g/L, neutrophils 110/μl, eosinophils 0/μl, platelets 4000/μl) with fatty marrow (Figure 1A: hematoxylin–eosin stain, ×4 objective). He had a history of bronchial asthma for 3 years and had been treated with inhaled corticosteroid in combination with long‐acting β_2_‐agonist (budesonide/formoterol fumarate). In addition, he had developed eosinophilic pneumonia requiring systemic administration of prednisolone (<30 mg/day) twice in the last 1.5 years. His family history was not significant for any serious diseases. Treatment with antithymocyte globulin, eltrombopag olamine, and cyclosporine (CsA) (500 mg/day) eliminated the need for blood transfusions and the CsA dose was gradually reduced. While pancytopenia continued (hemoglobin <97 g/L, neutrophils <1400/μl, platelets <30,000/μl), eosinophilia (>500/μl) appeared 17 months after the start of treatment. This increased to 4550/μl in 4 months after discontinuation of CsA treatment at 33 months. In addition, he presented with a rash with purpura on the legs, for which prednisolone (<20 mg/day) was not effective. Bone marrow examination showed mild hypocellular marrow with eosinophil expansion (43.8%) (Figure 1B: hematoxylin–eosin stain, ×4 objective; Figure 1C: hematoxylin–eosin stain, ×40 objective; Figure 1D: direct Fast Scarlet stain, ×40 objective). Increased blasts and significant dysplasia were not observed. G‐banding and fluorescence in situ hybridization for the *FIP1L1–PDGFRA* re‐arrangement detected no chromosomal aberrations. Moreover, skin biopsy revealed eosinophilic vasculitis and panniculitis, compatible with eosinophilic granulomatosis with polyangiitis (Figure 1E: hematoxylin–eosin stain, ×20 objective; Figure 1F: direct Fast Scarlet stain, ×40 objective). After re‐administration of CsA (150 mg/day), eosinophilia and skin lesions disappeared in 5 weeks.

**FIGURE 1:**
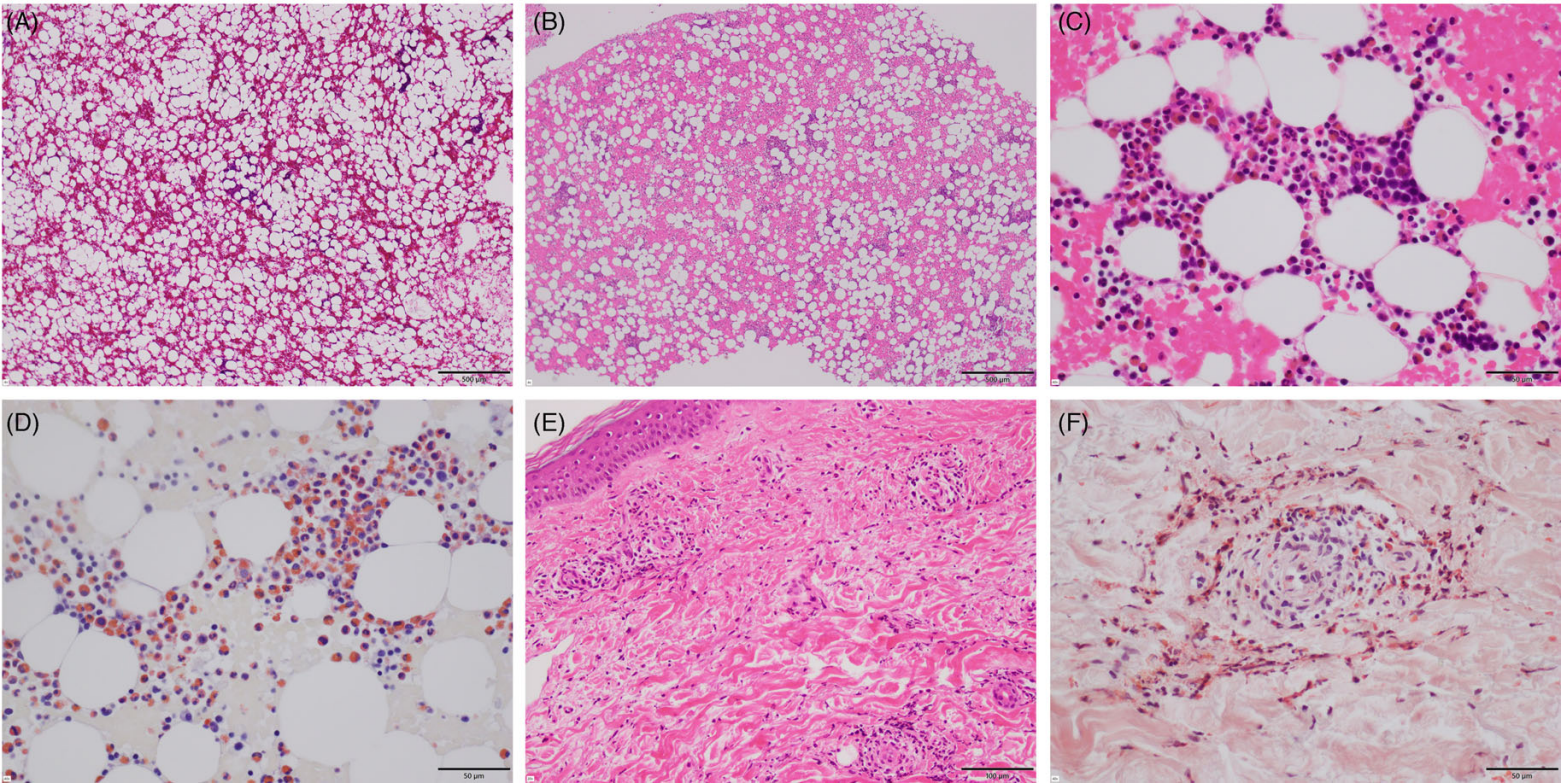
Bone marrow features (A–D) and histology of a skin biopsy specimen (E and F). (A and B) At presentation of aplastic anemia. (C–F) Four months after discontinuation of cyclosporine A (CsA) treatment

This case indicates that massive reactive eosinophilia can occur even in AA patients with eosinophil‐associated diseases, especially during dose reduction, or after the discontinuation of CsA.

## CONFLICT OF INTEREST

The authors declare that there is no conflict of interest.

## AUTHOR CONTRIBUTIONS

Aya Takahashi and Tsutomu Shinohara designed the research, drafted the manuscript, and created the figure. Aya Takahashi performed the skin biopsy. Yoshihito Iwahara, Aya Takahashi, Hisanori Machida, Keishi Naruse, Eiji Takeuchi, and Tsutomu Shinohara analyzed the data. Keishi Naruse contributed to pathological assessment. All authors revised the manuscript critically and approved the final manuscript.

